# Canine intestinal organoids as a platform for studying MHC class II expression in epithelial cells

**DOI:** 10.1186/s12860-025-00536-w

**Published:** 2025-04-08

**Authors:** Meg Nakazawa, Itsuma Nagao, Yoko M. Ambrosini

**Affiliations:** 1 Department of Veterinary Clinical Sciences, College of Veterinary Medicine, Washington State University, Pullman, Washington USA; 2 Department of Veterinary Internal Medicine, Graduate School of Agricultural and Life Sciences, The University of Tokyo, Tokyo, Japan

**Keywords:** Canine, Intestinal epithelial cells, Intestinal organoids, MHC class II, IFN-γ, Translational medicine, Comparative medicine

## Abstract

**Backgrounds:**

The interplay between intestinal epithelial cells (IECs), the immune system, and the gut microbiome is pivotal for maintaining gastrointestinal homeostasis and mediating responses to ingested antigens. IECs, capable of expressing Major Histocompatibility Complex (MHC) class II molecules, are essential in modulating immune responses, especially CD4 + T cells, in both physiological and pathological contexts. The expression of MHC class II on IECs, regulated by the class II transactivator (CIITA) and inducible by cytokine IFN-γ, has been traditionally associated with professional antigen-presenting cells but is now recognized in the context of inflammatory conditions such as inflammatory bowel disease (IBD). In veterinary medicine, particularly among canine populations, MHC (or Dog Leukocyte Antigen, DLA) expression on IECs underlines its significance in intestinal immune pathologies, yet remains underexplored. This study aims to leverage canine intestinal organoids as a novel in vitro model to elucidate MHC class II expression dynamics and their implications in immune-mediated gastrointestinal diseases, bridging the gap between basic research and clinical application in canine health.

**Results:**

Canine colonoids derived from healthy dogs showed significant expression of MHC class II and its promoter gene, *CIITA*, after IFN-γ treatment. This MHC class II induction was even more pronounced in differentiated colonoids cultured in Wnt-3a-depleted medium.

**Conclusions:**

This study provides insights into the role of IECs as antigen-presenting cells and demonstrates the use of intestinal organoids for investigating epithelial immune responses in inflammatory conditions.

**Supplementary Information:**

The online version contains supplementary material available at 10.1186/s12860-025-00536-w.

## Background

Intestinal epithelial cells (IECs) serve a critical role in the intricate dialogue among the immune system [1], the gut microbiome [2], and ingested antigens [3]. These cells have the capability to express Major Histocompatibility Complex (MHC) class II molecules [4], which are pivotal during both normal and inflammatory states [5], potentially influencing the immune responses of CD4 + T cells to gut antigens [6]. The regulation of MHC class II on non-hematopoietic cells like IECs is mediated by the class II transactivator (CIITA) and can be significantly induced by the cytokine IFN-γ [7]. Although MHC class II molecules are traditionally noted for their expression on professional antigen-presenting cells, including macrophages, B lymphocytes, and dendritic cells, recent evidence suggests that IECs also modulate MHC class II expression [8]. This modulation is particularly evident in the presence of inflammatory cytokines and in pathological states such as inflammatory bowel disease (IBD), where IFN-γ is known to stimulate such expression [9]. This regulation has been observed in vitro in various models, spanning from mice to humans [10, 11], highlighting a conserved mechanism of immune activation and regulation within the gut.

In veterinary medicine, specifically within canine populations, MHC—also known as the Dog Leukocyte Antigen (DLA)—is implicated in a spectrum of intestinal immune pathologies in breed-specific diseases such as IBD in German Shepherds and French Bulldogs [12, 13]. Notwithstanding, the literature on MHC class II expression in canine intestinal epithelial cells under normal and inflammatory conditions remains scarce. Evidence from studies indicates variable expression patterns, from constitutive presence in healthy tissues [14] to upregulated levels associated with diseases such as boxer colitis [15].

Despite the relevance of these findings, the exact role of MHC class II expression within canine intestinal epithelial cells remains inadequately characterized, creating a compelling case for in vitro models that could elucidate these dynamics. The lack of canine-derived cell lines and the limitations presented by murine models—which fail to fully recapitulate the diet and environmental conditions of humans and dogs [16]—underscore the necessity for alternative research models.

To address this gap, intestinal organoids have emerged as a promising tool [17]. Originating from endoscopic biopsies, these organoids are complex multicellular entities that mimic the diverse cellular makeup of the intestinal epithelium. They encompass a variety of epithelial cell types, such as enterocytes, goblet cells, tuft cells, and enteroendocrine cells [18]. One of the advantages of using intestinal organoids is the ability to derive them from both healthy and diseased tissues, including those from dogs afflicted with conditions like IBD [19]. Their capacity to co-culture with both bacteria [20] and immune cells [21] presents a robust platform for studying host-microbiota interactions. Considering the genetic polymorphism inherent to the MHC gene complex, case-specific organoid cultures offer a tailored approach to dissect the genetic underpinnings of MHC class II-associated pathologies.

This study investigates the utility of canine intestinal organoids as a representative in vitro platform for probing MHC class II expression and its implications in immune-mediated gastrointestinal diseases, providing a bridge between basic research and clinical application in canine health.

## Materials and methods

### Donor recruitment and intestinal sample collection

Colonic biopsy tissue samples were obtained from three clinically healthy canines during dental procedures at the Washington State University Veterinary Teaching Hospital Community Service. These dogs, aged between 1 and 12 years, were selected based on physical examinations and blood tests, and had no history of chronic diseases affecting major organs such as the heart, liver, kidneys, or intestines. Only dogs considered suitable for elective anesthesia were included in this study. Detailed information about the donor dogs is provided in Supplementary Table 1. The research was approved and carried out with the endorsement of the Washington State University IACUC, under ASAF#6993.

### Generation and maintenance of canine colonoids

The isolation of colonic crypts from biopsy samples was conducted using a method modified from a previous report [19]. In brief, colonic biopsy samples were washed in ice-cold Dulbecco’s phosphate-buffered saline (PBS, Thermo Fisher Scientific) with 1x Penicillin/Streptomycin (Thermo Fisher Scientific) five times. The tissue samples were minced with scissors and incubated in 12 mL of 30 mM EDTA solution (Thermo Fisher Scientific) in a 15 mL conical tube at 4 °C for 30 min to isolate crypts. After incubation in 30 mM EDTA for 30 min, the conical tube was left to stand for a few minutes, allowing tissue fragments to settle. The crypt-containing supernatant was then transferred to a new conical tube and centrifuged at 200 × g at 4 °C for 5 min, yielding approximately 150–240 crypts per tube. The isolated crypts were then resuspended in Matrigel (Corning) and plated in 48-well culture plates (Thermo Fisher Scientific), with approximately 30–40 crypts per 30 μL Matrigel drop. After Matrigel solidifying in a 37 °C incubator for 10 min, 300 µL of organoid expansion medium (EM) was added to each well. The EM composition was based on a previous study [19] and contained DMEM/F12 (Thermo Fisher Scientific) supplemented with 2 mM GultaMAX (Thermo Fisher Scientific), 10 mM HEPES (Thermo Fisher Scientific), 1x Penicillin/Streptomycin (Thermo Fisher Scientific), 10% (vol/vol) conditioned medium of Noggin [22], 20% (vol/vol) conditioned medium of R-spondin, 100 ng/mL recombinant murine Wnt-3a (PeproTech), 50 ng/mL murine Epidermal Growth Factor (EGF) (PeproTech), 10 nM Gastrin (Sigma-Aldrich), 500 nM A-83-01 (Sigma-Aldrich), 10 µM SB202190 (Sigma-Aldrich), 1 mM N-Acetyl-L-Cysteine (MP Biomedicals), 10 mM Nicotinamide (Sigma-Aldrich), 1x B27 supplement (Thermo Fisher Scientific), 1x N-2 MAX media supplement (R&D Systems), 100 µg/mL Primocin (Invivogen). The medium was refreshed every other day. For the first two days post-crypt isolation, 10 µM Y-27632 (Stem Cell Technologies) and 2.5 µM CHIR 99021 (Stem Cell Technologies) were included in the medium. The colonoids were passaged every 6–8 days. To dissolve the Matrigel domes, Cell Recovery Solution (Corning) were used at 4 °C for 30 min. The colonoids were then disrupted using TrypLE Express (Thermo Fisher Scientific), centrifuged, and resuspended in Matrigel for re-seeding in a 48-well plate at a 1:6–8 dilution.

### Canine colonoids differentiation

The colonoids were cultured in the EM for 2 days to obtain a stable number of mature colonoids before inducing colonoids differentiation based on previous human studies [23]. After two days, the EM was completely removed from each well, and the Matrigel dome containing canine colonoids was washed with PBS twice, following repletion with 300 μL of differentiation medium (DM). The composition of DM was based on previous study [24], which was the EM without Wnt-3a, nicotinamide, or SB202190. DM was refreshed every two days and colonoids were cultured in DM for 4 days. For comparison analysis, colonoids continuously cultured in EM total of 6 days were used as a control.

### Cytokine treatment of canine colonoids

To determine whether proinflammatory cytokines induce MHC class II expression in canine colonoids, we selected three major proinflammatory cytokines namely IFN-γ, TNF-α, and IL-1β that are closely related to human and canine intestinal inflammation, including IBD [25–27]. Canine colonoids were initially cultured in EM for 2 days, followed by a medium change to differentiation medium (DM). On day 4, colonoids were treated with 30 ng/mL of recombinant canine IFN-γ, TNF-α, or IL-1β (R&D Systems) in either EM or DM. To further investigate the dose- and time-dependent regulation of MHC class II expression by IFN-γ, colonoids were treated with 30 or 60 ng/mL of recombinant canine IFN-γ in EM or DM after 2 days of EM culture followed by 2 days in DM. Each cytokine was dissolved in PBS according to the manufacturer’s instructions and the same amount of PBS was added to the medium as a vehicle control. The concentration of these proinflammatory cytokines was selected based on previous studies [28–30].

### Quantitative real-time RT-PCR

Total RNA was isolated from the colonoids at several timepoints utilizing the RNeasy Mini Kit (Qiagen). Individual RNA concentration was determined using Nanodrop One C (Thermo Fisher Scientific). 1 μg of RNA was served for the synthesis of complementary DNA using the High-Capacity cDNA Reverse Transcription Kit (Thermo Fisher Scientific). To evaluate the differentiation status, we quantified a variety of multilineage marker genes, Chromogranin A (*CgA*) for enteroendocrine cells, Lysozyme (*Lyz*) for Paneth cells and Mucin 2 (*MUC2*) for goblet cells based on previous studies [19, 23, 31]. To compare the effects of three inflammatory cytokines, we measured the expression of dog leukocyte antigen DR alpha (*DLA-DRA*), a highly expressed MHC class II gene with no genetic polymorphism, in colonoids 24 h after cytokine treatment [32]. For evaluation the impact of IFN-γ treatment on MHC class II expression, *DLA-DRA* and Class II transactivator (*CIITA*) were quantified using the colonoids at 0, 24 and 48 h after IFN-γ exposure [33]. The gene expression was quantified through PowerUp SYBR Green Master Mix (Thermo Fisher Scientific) on a CFX96 Touch Real-Time PCR Detection System (Bio-Rad). The following thermal cycling conditions were applied: 2 min of UDG activation at 50°C and 2 min at 95°C followed by 40 cycles of 5 s of denaturation at 95°C, 30 s of annealing/extension at 60°C and a read step. After the completion of cycles, a melting curve was generated, by heating from 65 to 95°C, in increments of 0.5°C/5 s. RT-qPCR reactions, performed in duplicate, allowed for the calculation of relative gene expression based on previous study [34]. Internal controls for these reactions included *HMBS, HPRT1*, and *SDHA*, as previously reported [35]. Cytokine treatment was replicated three times, with approximately 100 colonoids used per well in each time point and experiment [36]. Gene expression analysis for *DLA-DRA* and *CIITA* was performed using three biological replicates (2 males, 1 female), while two biological replicates (1 male, 1 female) were used for differentiation marker genes. The details of all primers used in this study are provided in Supplementary Table 2.

### Peripheral blood mononuclear cells (PBMCs) extraction

For positive control for immunocytochemistry, PBMCs from single dog underwent bioscopy was used. The PBMCs were extracted using Histopaque 1077 (Sigma-Aldrich), following manufacturer instruction. In short, 5–10 mL of whole blood sample in EDTA tube (Becton Dickinson) was centrifuged at 800 x g at room temperature for 10 min. The buffy coat containing PBMCs diluted with PBS up to 3 mL was layered over 3 mL of Histopaque 1077 (Sigma-Aldrich) and centrifuged at 400 x g at room temperature (RT) for 30 min. After centrifugation, the layer containing the PBMCs was extracted to new 15 mL conical tube and diluted with PBS and centrifuged at 400 x g at RT for 10 min. After removal of the supernatant, the PBMCs were resuspended in 2 mL of ACK lysing buffer (Thermo Fisher Scientific) and incubated at RT for 10 min, followed by diluting with PBS and centrifuged once more.

### Immunocytochemistry

Colonoids were fixed using 4% paraformaldehyde (Thermo Fisher Scientific) for 15 min at room temperature. This was followed by permeabilization with 0.3% Triton X-100 (Thermo Fisher Scientific) for 15 min and blocking with 2% bovine serum albumin (BSA) (Cytiva) for an hour. The colonoids were then incubated with primary antibody: recombinant anti-HLA-DR antibody (TAL1B5, 1:100, Abcam), following treatment with goat anti-mouse IgG H&L (DyLight 488) (1:500, Abcam) for 1 h at RT. Nuclei were stained using DAPI (1:1000, Thermo Fisher Scientific) and F-actin was stained using Alexa Fluor™ 647 Phalloidin (1:250, Thermo Fisher Scientific). Following another PBS wash, the colonoids were suspended in Prolong Gold Antifade reagent (Thermo Fisher Scientific) and mounted on a glass bottom dish (Matsunami).

Canine PBMCs constitutively expressing MHC class II molecule were served as positive control. The PBMCs were attached to a glass bottom dish using the method described in the previous study [37] and stained in the same way as the colonoids. Canine colonoids treated with 60 ng/mL IFN-γ in DM for 48 h were incubated with a mouse IgG1 kappa isotype control antibody (P3.6.2.8.1, 1:50, Thermo Fisher Scientific) as the negative control instead of the primary antibody.

Fluorescence imaging was conducted using a white light point scanning confocal microscope (SP8-X, Leica), and image processing was done using LAS X software (Leica). For quantitative analysis of DLA-DR expression, we randomly selected ten to fifteen random fields of view from one or two biological replicates. ImageJ software was used for fluorescence image analysis [38]. DLA-DR expression was assessed by measuring the mean area intensity, and normalized by number of nuclei in the same image.

## Statistical analyses

Quantitative data in our study were processed using R v 3.4.1 (The R Foundation), and visualized using GraphPad Prism version 10.1.1 (GraphPad Software). We employed the Kruskal-Wallis test followed by a Dunn’s multiple comparison test for RT-qPCR and immunohistochemistry to compare the differentiation marker, *DLA-DRA* and *CIITA* gene expression and DLA-DR fluorescent intensity. Results are reported as mean ± standard error of the mean (SEM), and a p-value of 0.05 or less was considered to denote statistical significance.

## Results

### The differentiation induction of canine colonoids

**Fig. 1:**
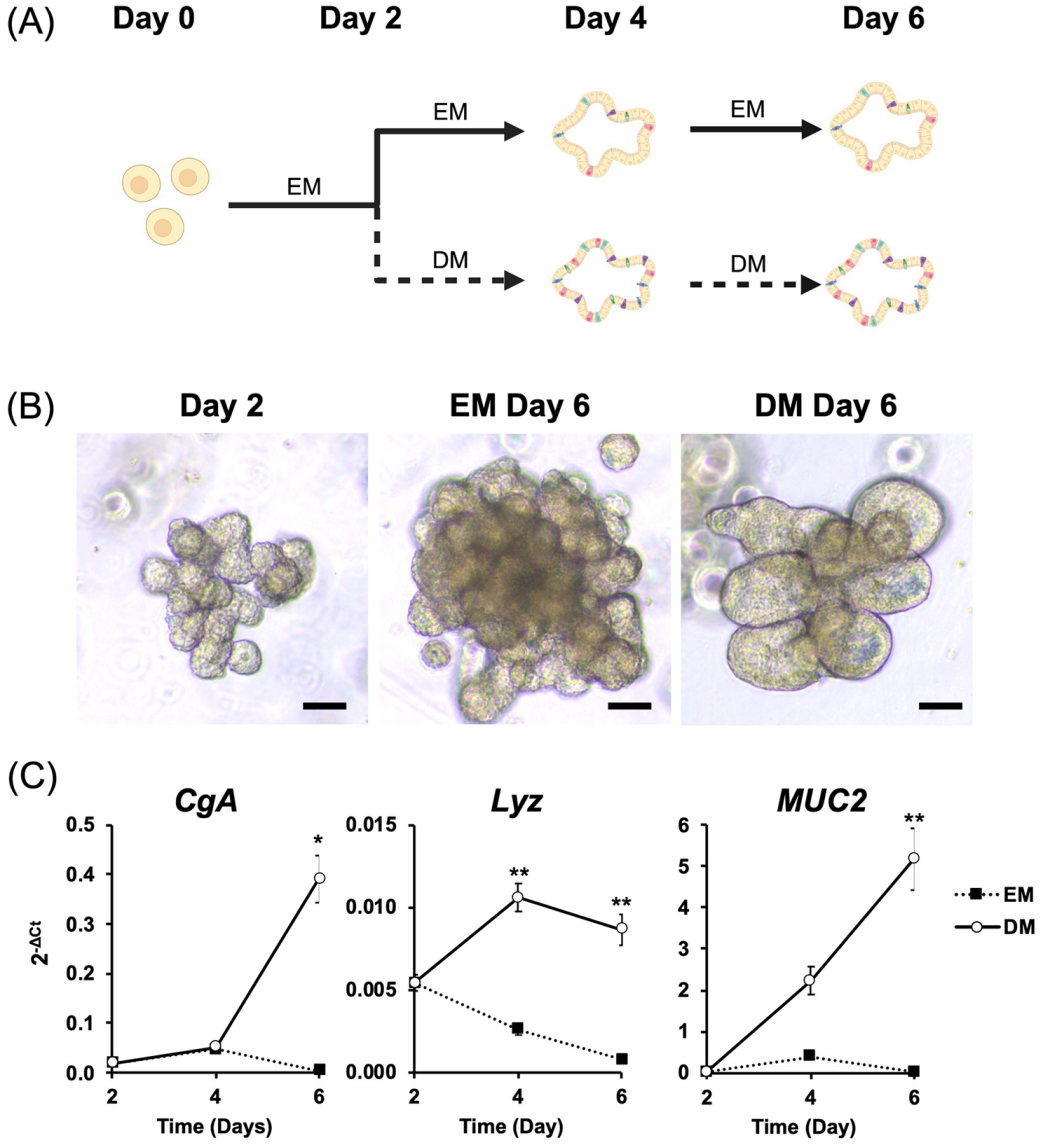
Differentiation of canine colonoids. (**A**) A schematic representation of the flow of the differentiation induction. The colored dots in the intestinal organoid represent specialized epithelial cells, like goblet and enteroendocrine cells, that differentiate from stem cells. The canine colonoids were initially cultured for 2 days in expansion medium (EM) followed by changing the medium from EM to differentiation medium (DM). For comparison group, colonoids cultured in EM total of 6 days were used. The colonoids were collected at day 2, 4 and 6 after passaging. This schematic was created with BioRender.com. (**B**) The representative image of the canine colonoids at day 2 and 6. Scale bar = 50 μm. (**C**) Three differentiation markers’, Chromogranin A (*CgA*), Lysozyme (*Lyz*) and Mucin 2 (*MUC2*), mRNA were quantified using RT-qPCR. Two biological replicates were used for this analysis and differentiation/non-differentiation treatment were replicated three times. The error bars represent the standard error of the mean (SEM). *p< 0.05, **p< 0.01

A schematic image depicting the flow of differentiation was shown in Fig. 1a. The colonoids in DM maintained a budding-like morphology similar to those in EM, with no apparent differences observed (Fig. 1b). We assessed the expression of differentiation markers, *CgA, Lyz* and *MUC2* between EM and DM at day 2, 4 and 6 after colonoid passaging using quantitative RT-PCR (Fig. 1c) to confirm the colonoid differentiation. *CgA* expression was significantly increased from day 4 to day 6 in DM compared with EM group (*p* = 0.017). A significant increase in *Lyz* expression was observed from day 2 to day 4, with significant differences compared to the EM group detected on days 4 and 6 (*p* = 0.002 for both). A time-dependent increase of *MUC2* expression was noted only in the DM group, contrasting to no significant change in the EM group (at day 6, *p* = 0.002).

### IFN-γ induced the *DLA-DRA* expression

**Fig. 2:**
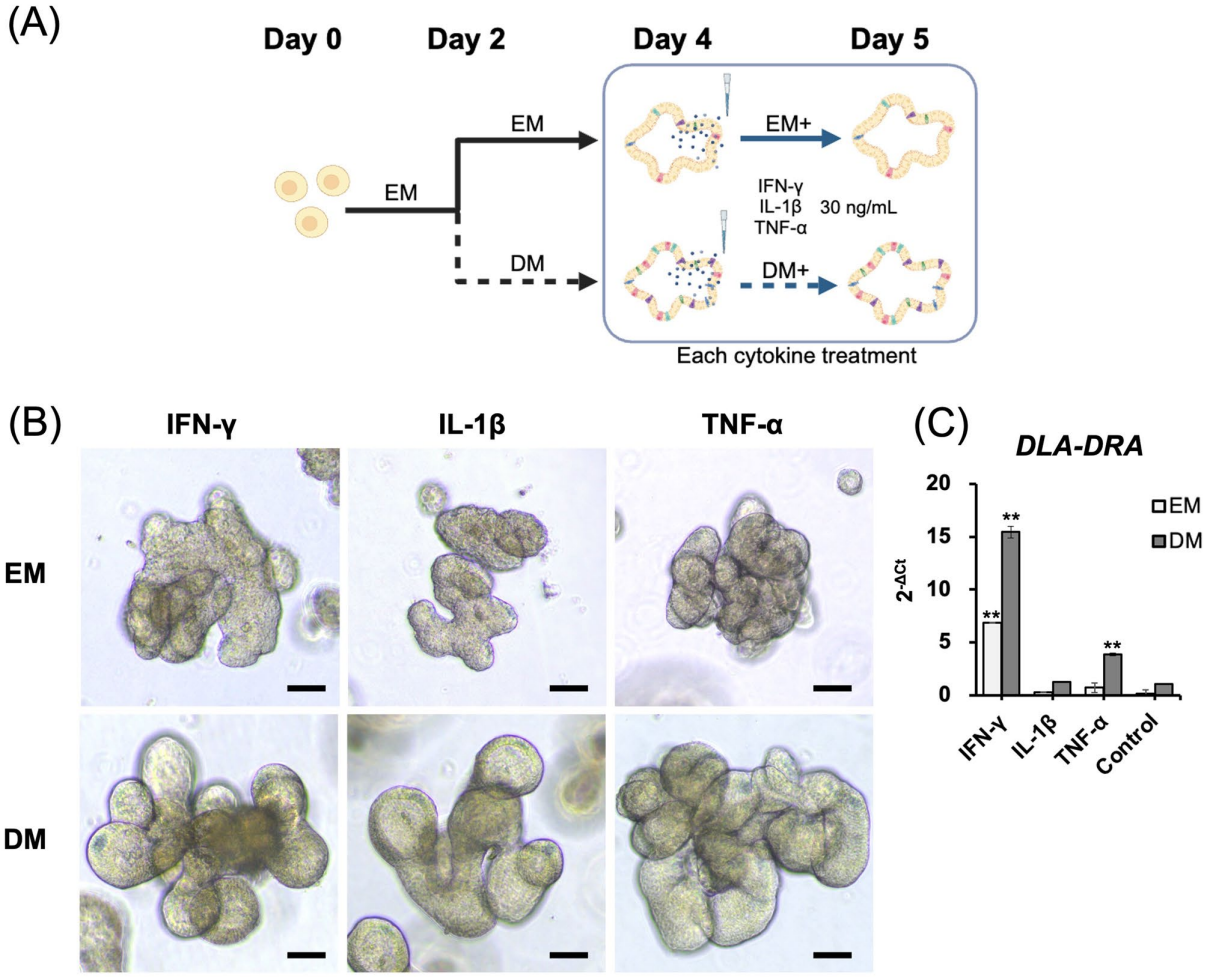
MHC class II gene expression in colonoids after treatment with the three major proinflammatory cytokines. (**A**) A schematic representation of the flow of cytokine treatments. The colonoids were initially cultured for 2 days in EM, followed by changing the medium from EM to DM. On day 4, colonoids were treated with 30 ng/mL of recombinant canine IFN-γ, TNF-α, or IL-1β in either EM or DM for 24 h. This schematic was created using BioRender.com. (**B**) The representative image of the canine colonoids at 24 h after treatment with 30 ng/mL of IFN-γ, IL-1β and TNF-α in EM and DM. Scale bar = 50 μm. (**C**) *DLA-DRA* expression was quantified using RT-qPCR 24 h after cytokine treatment (30 ng/mL IFN-γ, IL-1β, or TNF-α) in EM or DM. Three biological replicates were used for this analysis, with each cytokine treatment performed in triplicate. Error bars represent the SEM. **p < 0.01

Then we evaluate whether cytokines associated with intestinal inflammatory condition, such as IL-1β and TNF-α in addition to IFN-γ have potential to induce the *DLA-DRA* expression in our colonoids in EM and DM using RT-qPCR. A schematic image presenting the flow of each cytokine treatment workflow is shown in Fig. 2a. There were no significant morphological changes seen in each cytokine groups (Fig. 2b). *DLA-DRA* expression increased in colonoids cultured in EM following IFN-γ stimulation alone, while colonoids cultured in DM showed increased *DLA-DRA* expression with both IFN-γ and TNF-α stimulation. Regardless of the culture condition (EM vs. DM), IFN-γ induced the highest increase in *DLA-DRA* expression in canine colonoids (Fig. 2c).

**Fig. 3:**
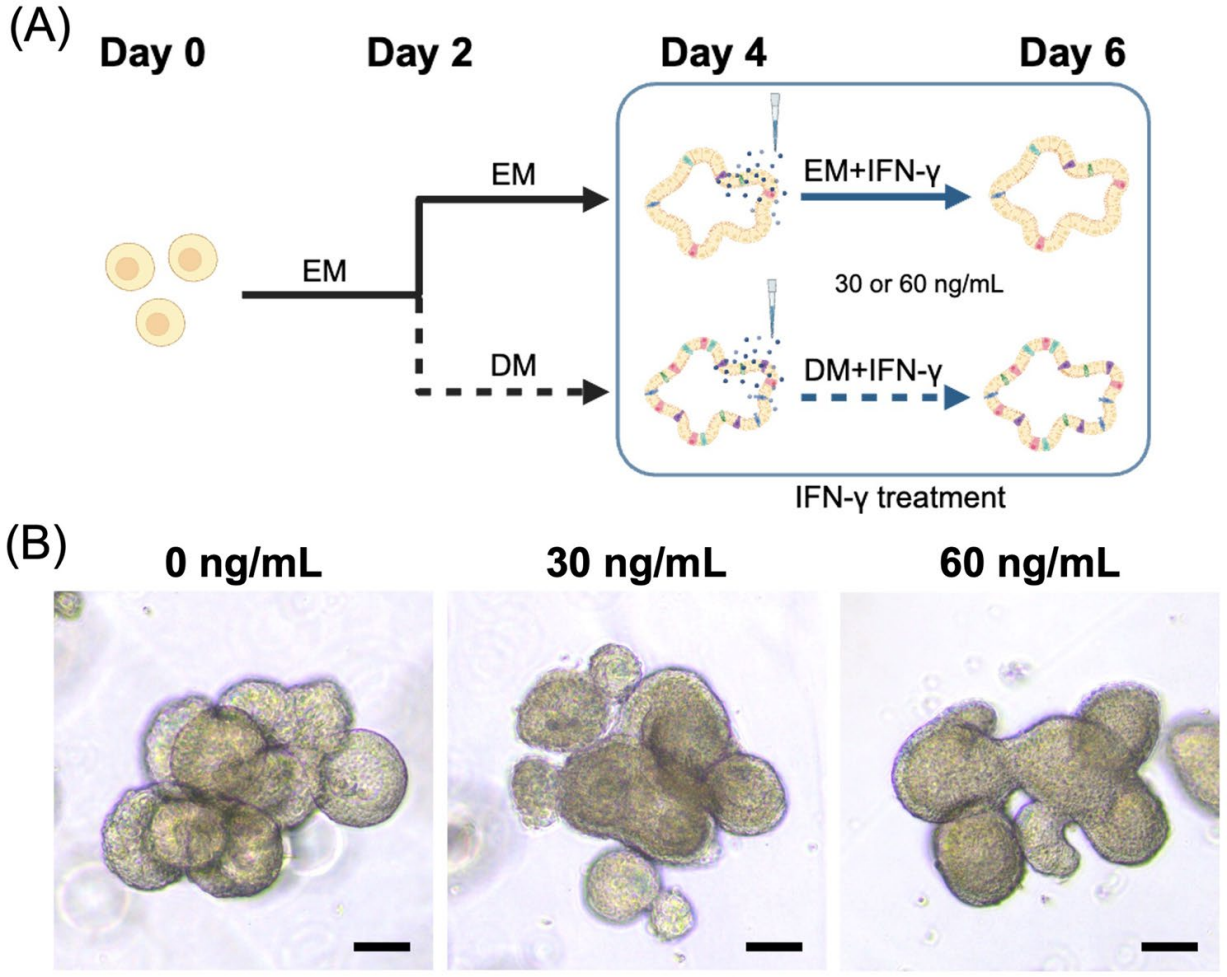
IFN-γ treatment of canine colonoids. (**A**) A schematic representation of the flow of the IFN-γ treatment. The colonoids initially cultured for 2 days in EM, followed by changing the medium from EM to DM. At day 4, the colonoids were treated with 0, 30 and 60 ng/mL of IFN-γ. For comparison, colonoids treated with IFN-γ in EM were used. The colonoids were then collected at 0, 24 and 48 h after IFN-γ treatment for further analysis. This schematic was created with BioRender.com. (**B**) The representative image of the canine colonoids in DM at day 6. Scale bar = 50 μm

**Fig. 4:**
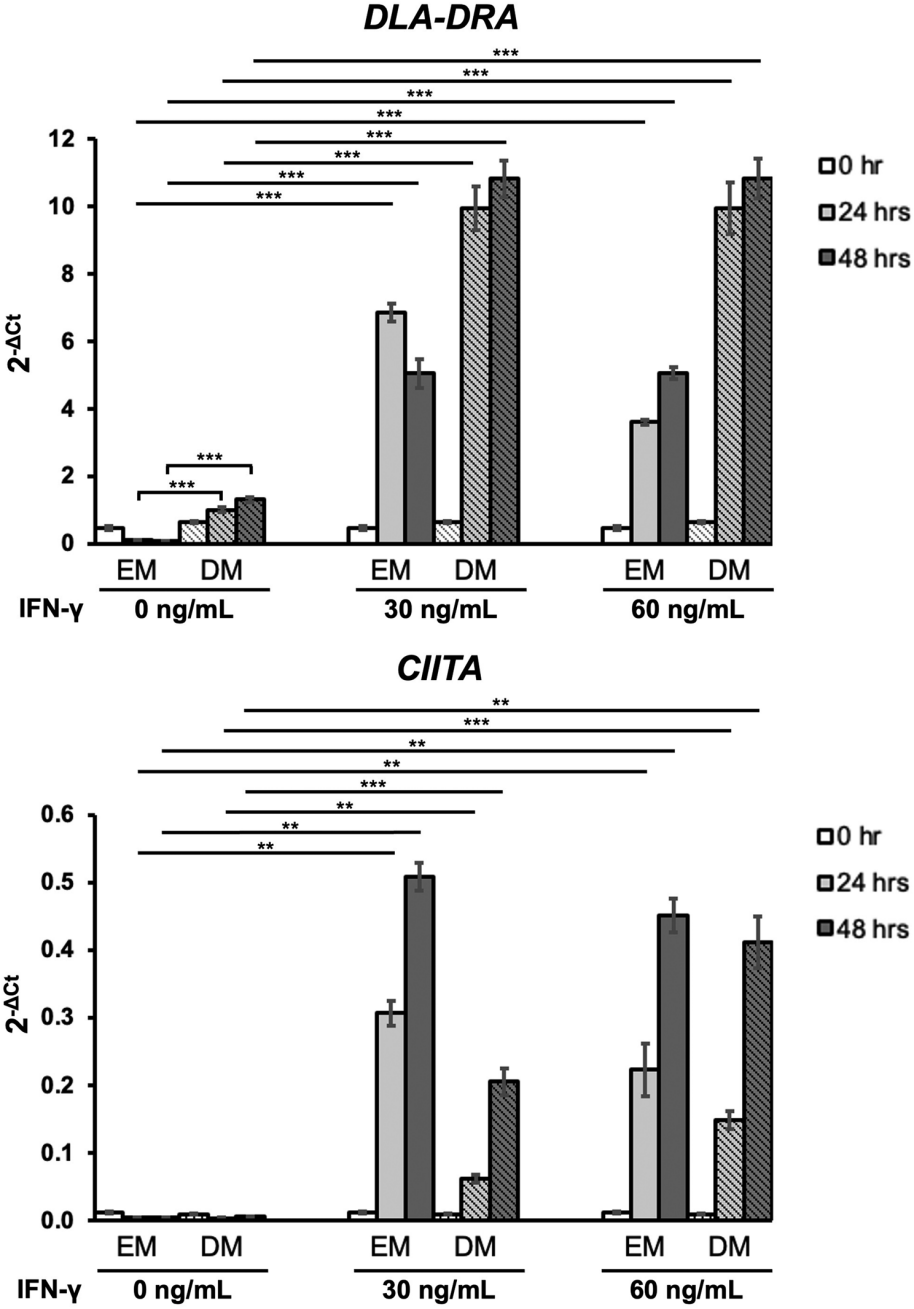
MHC class II related gene expression in colonoids after IFN-γ treatment. *DLA-DRA* and *CIITA* mRNA expression was assessed using RT-qPCR at 0, 24 and 48 h after IFN-γ treatment (0, 30 and 60 ng/mL) in EM and DM. Three biological replicates were used for this analysis and cytokine treatment was replicated three times. Significant differences between the EM and DM control groups (0 ng/mL) at 24 and 48 h are indicated with bracket-style markers. Significant differences between IFN-γ-treated groups (30 or 60 ng/mL) and their respective EM or DM control groups are indicated with flat line markers. The error bars represent the SEM. **p< 0.01, ***p< 0.001

A schematic image depicting the flow of IFN-γ treatment was shown in Fig. 3a. There were no significant morphological changes seen in each cytokine groups (Fig. 3b, Supplementary Fig. 1). We then assessed the *DLA-DRA* expression after treatment with IFN-γ (0, 30 and 60 ng/mL) at three timepoints (0, 24 and 48 h) (Fig. 4). The DM control group exhibited significantly higher *DLA-DRA* expression than the EM control group at 24 and 48 h (*p*< 0.001); however, this effect was modest compared to the strong upregulation observed with IFN-γ stimulation. There was an increase of *DLA-DRA* expression at 24 and 48 h after IFN-γ treatment with DM compared with non-treated DM group (both conc. *p* < 0.001). The EM group showed a slightly lower induction of IFN-γ induced-*DLA-DRA* expression than the DM group at both time points (*p*< 0.001). A time-dependent increase of *CIITA* expression was observed in both IFN-γ concentrations with DM at 24 h (IFN30 *p* = 0.002: IFN60 *p*< 0.001) and 48 h (IFN30 *p*< 0.001: IFN60 *p* = 0.008) after IFN-γ treatment. At 30 ng/mL of IFN-γ, *CIITA* expression in the EM group was significantly higher than in the DM group at both time points (*p*= 0.001), a difference not observed at 60 ng/mL.

### DLA-DR protein expression in canine colonoids

**Fig. 5:**
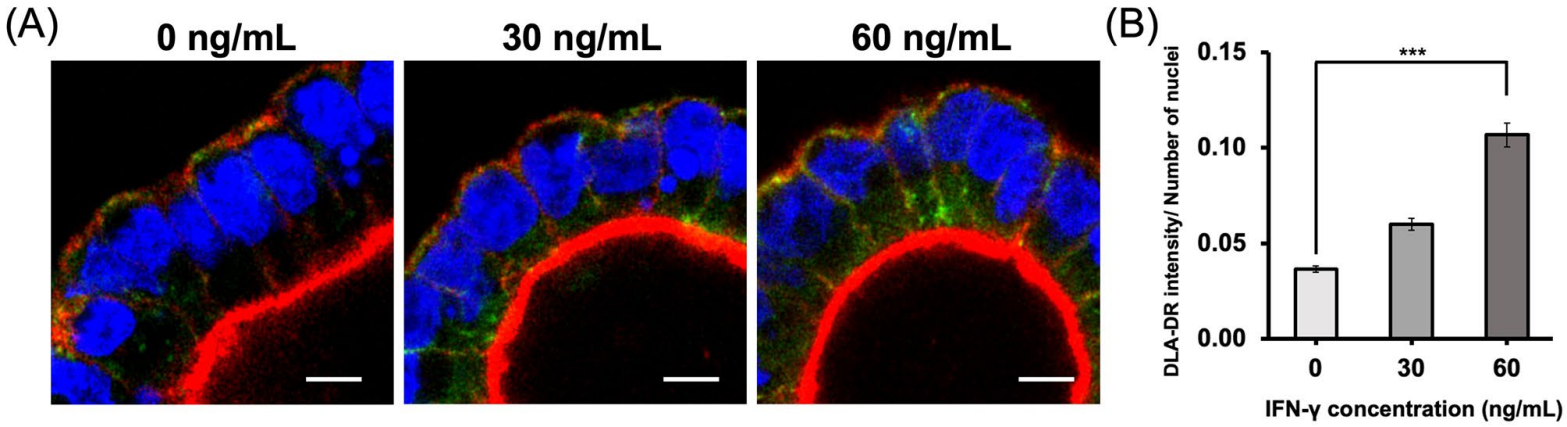
The effect of IFN-γ on MHC class II protein expression in colonoids. Impact of IFN-γ exposure on DLA-DR molecule expression. (**A**) Representative confocal images of colonoids 48 h after IFN-γ treatment (0, 30 and 60 ng/mL) in DM. DLA-DR (green), nuclei (DAPI, blue) and F-actin (red) were stained. Scale bar = 5 µm. (**B**) Comparative analysis of mean intensity of DLA-DR at 48 h after IFN-γ treatment (0, 30 and 60 ng/mL) in DM. Fifteen fields of view were randomly selected from two biological replicates. Mean intensity was normalized using number of nuclei in the same image. The error bars represent the SEM. ***p< 0.001

The images of positive control (PBMCs) and negative control (colonoids with isotype control antibody) for this study were shown in Supplementary Fig. 2. We evaluate the DLA-DR protein expression using the colonoids 48 h after IFN-γ treatments (0, 30 and 60 ng/mL) (Fig. 5a). A dose-dependent increase of DLA-DR intensity was noted (Fig. 5b) and significant difference was seen between 0 and 60 ng/mL of IFN-γ treatment (*p* = 0.001). In EM condition the significant difference also was seen between 0 and 60 ng/mL of IFN-γ treatment (*p* = 0.021) (Supplementary Fig. 3).

## Discussion

Our study represents a significant stride in the understanding of canine immune mechanisms, demonstrating that DLA-DR expression can be induced in colonoids derived from canine endoscopic biopsy samples with IFN-γ stimulation. This induction underscores the importance of MHC class II expression by IECs beyond classical antigen-presenting cells. IECs, acting as non-traditional antigen-presenting cells, have an emerging role in modulating immune responses within the gut’s unique environment. The expression of MHC class II on these cells could thus be pivotal in the immune surveillance of the intestinal epithelium and in shaping the local immune landscape.

Our initial exploration focused on determining whether organoids differentiate into functional epithelial cells more effectively when cultured in a DM, which lacks Wnt-3a, nicotinamide, and SB202190, compared to an EM rich in Wnt-3a, nicotinamide, and SB202190. This is based on the hypothesis that organoids grown in an EM might not accurately mimic the in vivo conditions of IECs as those cultured in a DM would. Our findings confirm that organoids cultured in DM indeed undergo differentiation into functional epithelial cells. Future studies could explore lineage commitment and fate-determining processes in canine intestinal epithelial cells in more detail. It is important to note that no obvious morphological changes were observed in response to different cytokine treatments. Further investigations could utilize 2D monolayers derived from 3D canine organoids to assess morphological changes more quantitatively. However, our previous study [39] demonstrated that inflammatory cytokines influence tight junction expression and the regenerative capacity of stem cells. Therefore, it is likely that cytokine treatment contributed to a reduction in epithelial barrier function and an increase in permeability.

Interestingly, prior research with human intestinal organoids demonstrated that no significant increase in MHC class II protein levels was observed with DM alone; however, a moderate increase in mRNA levels was noted [10]. This suggests that IECs that have undergone differentiation may possess a heightened capacity to express MHC class II in response to IFN-γ [10]. Our findings corroborate this observation, showing that organoids achieve the highest levels of MHC class II expression when treated with IFN-γ in a DM, compared to treatment in DM alone or with IFN-γ in an EM. The observed differences in *CIITA* and *DLA-DRA* expression between EM- and DM-cultured colonoids (Fig. 4) raise intriguing questions about the factors governing MHC class II regulation in intestinal epithelial cells. It is possible that certain cytokine-mediated pathways differentially affect CIITA-dependent and CIITA-independent MHC class II expression, as reported in other antigen-presenting cells [40]. While our study establishes canine colonoids as a model for studying MHC class II expression, further investigations are needed to explore the specific mechanisms driving these disparities.

*DLA-DRA* and *CIITA* expression patterns in the EM group differed from those in the DM group, suggesting distinct cellular activation states under different culture conditions (Fig. 4). Wnt-3a in EM maintains the undifferentiated state of crypt progenitors [23, 24], and MHC class II expressed in intestinal stem cell populations is involved in intestinal epithelial remodelling [41], suggesting that undifferentiated organoids may exhibit different behaviour of MHC class II expression. This underscores the importance of both the differentiation status of IECs and the specific stimuli they are exposed to in modulating MHC class II expression. This indicates that differentiated IECs are likely more competent at expressing MHC class II in response to IFN-γ, highlighting the critical role of the culture medium in determining the functional capabilities of epithelial cells derived from organoids. Further investigation is warranted to elucidate the mechanisms by which DM promotes MHC class II expression, including the analysis of cytokine receptor expression in EM and DM-cultured organoids, as well as the effects of IL-1β and TNF-α stimulation in DM.

The relevance of MHC class II expression extends to the pathogenesis of IBD, both in humans and canines. Conditions such as Boxer colitis mirror aspects of very early onset IBD in human [42], suggesting that a common immunological basis may underlie these diseases. Similarly, the link between MHC class II and celiac disease in humans [43] hints at the complex interplay between genetic susceptibility, antigen presentation, and immune response—parallels that may hold true in canine counterparts.

MHC class II molecules are essential for antigen presentation and immune regulation, with different MHC class II types exhibiting varying expression levels and responsiveness to cytokine stimulation. *DLA-DRA* was selected as the target gene for this study due to its consistent expression and lack of genetic variation. This selection is consistent with previous studies that have used *DLA-DRA* as a marker for assessing MHC class II expression [44]. Future studies incorporating sequencing analyses and broader MHC class II profiling could further expand these findings, providing a more comprehensive understanding of the genetic and functional regulation of antigen presentation in the canine intestinal epithelium.

Our findings using canine colonoids expand our understanding of IECs as antigen-presenting cells. Combining these findings with our canine colonoid-derived monolayers [31, 45] provides a platform for co-culture with immune cells, such as T cells and macrophages, as well as exposure to antigens, including bacteria [21, 46–48]. Future experiments could leverage this system to investigate the functional consequences of IEC-mediated antigen presentation, including its effects on T cell activation, cytokine production, and immune response regulation. Further studies incorporating protein-level analysis and broader DLA typing could provide deeper insights into the lineage-specific effects of CIITA and its role in regulating MHC class II expression in canine colonoids. However, given the limitations of available antibodies for staining DLA proteins, RNA *in situ* hybridization (RNA-ISH) could serve as an alternative approach to co-localize DLA expression with specific epithelial cell lineages, providing valuable insights into the cellular context of MHC class II regulation.

However, while in vitro organoid cultures offer a controlled environment to dissect cellular and molecular interactions, they fall short of fully replicating the intricate and dynamic in vivo milieu. The absence of in vivo data signifies a limitation of our study, underscoring the necessity for complementary research that integrates live animal models to confirm our findings and to more comprehensively understand the systemic implications of MHC class II expression in canine IBD. Advanced in vitro platforms such as ‘gut-on-a-chip’ technology may bridge this gap, providing a dynamic, in vivo-like environment capable of supporting co-culture systems with a variety of cell types, including immune cells [49]. This technological leap may afford us deeper insights into MHC class II’s role and enable more sophisticated explorations into the immunological interplay at play in IBD, within a controllable yet complex in vitro setting.

## Conclusion

In conclusion, this study expands our understanding of IECs as antigen-presenting cells and highlights the potential of intestinal organoids as a model for investigating the immunogenetics of colitis. Since dogs develop intestinal inflammatory conditions similar to those in humans, canine models and organoids serve as valuable tools for advancing our knowledge and improving treatment strategies for this complex disease.

## Electronic supplementary material

Below is the link to the electronic supplementary material.

Supplementary Material 1

## Data Availability

The datasets generated and analyzed during the current study are available from the corresponding author upon reasonable request.

## Abbreviations

CgA: Chromogranin A
CIITA: Class II transactivator
DM: Differentiation medium
EM: Expansion medium
IBD: Inflammatory bowel disease
EM: Expansion medium
IECs: Intestinal epithelial cells
Lyz: Lysozyme
MHC: Major Histocompatibility Complex
MUC2: Mucin 2
PBMCs: Peripheral blood mononuclear cells
