# Supplementary material for: Canine intestinal organoids as a platform for studying MHC class II expression in epithelial cells
Source: BMC Mol Cell Biol. 2025 Apr 8;26:11. doi: 10.1186/s12860-025-00536-w (PMC11980282; doi:10.1186/s12860-025-00536-w)
Supplement: Supplementary file 1 — Supplementary Material 1 [file 12860_2025_536_MOESM1_ESM.docx]

**Supplementary Material**

**Supplementary Table 1. Signalment of healthy dogs included this study.** A summary table of the donor information with sample name, breed, sex and age.


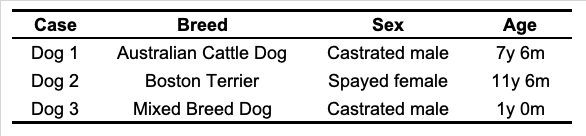


**Supplementary Table 2. Primer information.** Gene name, forward (F) and reverse (R) sequences, product size and GenBank accession number are listed.

**
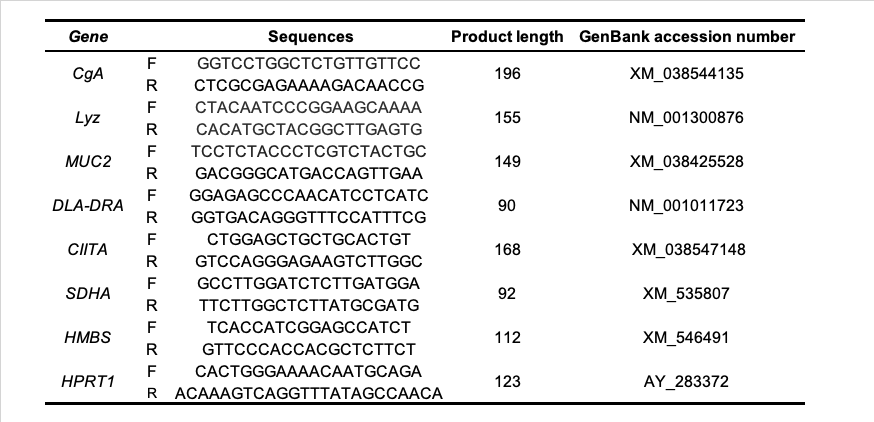
**


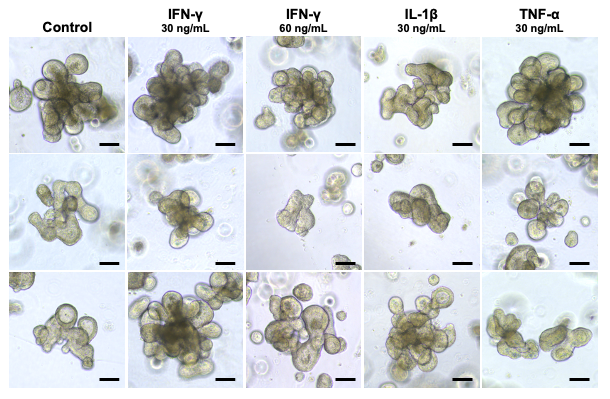


**Supplementary Figure 1.** **The representative image of the canine colonoids in DM at day 5.** Canine colonoids were initially cultured in EM for 2 days, followed by a medium change to differentiation medium (DM). On day 4, colonoids were treated with 30 ng/mL of recombinant canine IFN-γ, TNF-α, or IL-1β or 60 ng/mL of IFN-γ in DM for 24 hours. The control group was treated with DM without cytokines for the same duration. Scale bar = 100 μm.

**Supplementary Figure 2. Positive and negative controls for DLA-DR immunocytochemistry.** (A) Canine peripheral blood mononuclear cells served as the positive control. (B) Canine colonoids treated with an isotype control antibody instead of the primary antibody served as the negative control. Green is DLA-DR, blue is DAPI and red is F-actin. Scale bar = 10 μm.


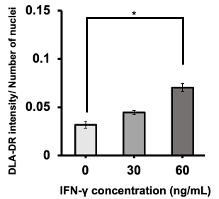


**Supplementary Figure 3. Influence of IFN-γ in expansion medium (EM) on DLA-DR expression.** Comparison of mean DLA-DR intensity at different IFN-γ concentrations in EM 48 hours after IFN-γ treatment. DLA-DR intensity was normalized using the number of nuclei in the same image. Ten fields of view were randomly selected from one organoid line. The error bars represent the standard error of the mean (SEM). **p*<0.05.
